# Push hard, push fast: quasi-experimental study on the capacity of elementary schoolchildren to perform cardiopulmonary resuscitation

**DOI:** 10.1186/1757-7241-21-41

**Published:** 2013-05-21

**Authors:** Simon Berthelot, Miville Plourde, Isabelle Bertrand, Amélie Bourassa, Marie-Maud Couture, Élyse Berger-Pelletier, Maude St-Onge, Renaud Leroux, Natalie Le Sage, Stéphanie Camden

**Affiliations:** 1 Department of Emergency Medicine, CHU de Québec – CHUL, 2705 Boul. Laurier, Québec, Qc G1V 4G2, Canada; 2 CHU de Québec - HSFA, Québec, Qc, Canada; 3 CHU de Québec – HSS, Québec, Qc, Canada; 4 CHAUQ - Hôtel-Dieu de Lévis, Québec, Qc, Canada; 5 Centre de recherche du CHU de Québec, Québec, Qc, Canada

**Keywords:** Child, Cardiopulmonary resuscitation/standards, Cardiopulmonary resuscitation/methods, Education, School health services, Age factors

## Abstract

**Background:**

The optimal age to begin CPR training is a matter of debate. This study aims to determine if elementary schoolchildren have the capacity to administer CPR efficiently.

**Methods:**

This quasi-experimental study took place in a Quebec City school. Eighty-two children 10 to 12 years old received a 6-hour CPR course based on the American Heart Association (AHA) Guidelines. A comparison group of 20 adults who had taken the same CPR course was recruited. After training, participants’ performance was evaluated using a Skillreporter manikin. The primary outcome was depth of compressions. The secondary outcomes were compression rate, insufflation volume and adherence to the CPR sequence. Children’s performance was primarily evaluated based on the 2005 AHA standards and secondarily compared to the adults’ performance.

**Results:**

Schoolchildren did not reach the lower thresholds for depth (28.1 +/− 5.9 vs 38 mm; one-sided p = 1.0). The volume of the recorded insufflations was sufficient (558.6 +/222.8 vs 500 ml; one-sided p = 0.02), but there were a significant number of unsuccessful insufflation attempts not captured by the Skillreporter. The children reached the minimal threshold for rate (113.9 +/−18.3 vs 90/min; one-sided p < 0.001). They did not perform as well as the adults regarding compression depth (p < 0.001), but were comparable for insufflation volume (p = 0.83) and CPR sequence.

**Conclusions:**

In this study, schoolchildren aged 10–12 years old did not achieve the standards for compression depth, but achieved adequate compression rate and CPR sequence. When attempts were successful at generating airflow in the Skillreporter, insufflation volume was also adequate.

## Background

Between one third and one half of out-of-hospital cardiac arrests in Canada are witnessed by a bystander [1]. With early access to EMS, bystander cardiopulmonary resuscitation (CPR) is the most important factor in predicting successful outcome for these patients, associated with a nearly four-fold increase in the odds of surviving [2]. While reported to be as high as 30% in the Seattle area where CPR knowledge is widespread, out-of-hospital cardiac arrest survival rate in Canada ranges from 3.2 to 6.7% [1,3,4]. This stresses the importance of strengthening the second link of the survival chain through increased bystander CPR knowledge in Canadian communities.

Implementing CPR education within the school curriculum has been proposed as a solution to CPR knowledge diffusion [5-9]. However, the optimal age to begin such training is unclear [5,7,10]. Few studies have addressed whether elementary schoolchildren can adequately learn and perform CPR, but none have provided a comprehensive assessment of all CPR skills [5,10-14]. Consequently, the objective of this study was to determine if children 10 to 12 years of age have the capacity to administer CPR to an adult. More specifically, our primary objective was to assess if children can achieve the minimum requirements for compression depth, compression rate, and insufflation volume, as well as perform the correct CPR sequence of actions as outlined by the 2005 American Heart Association (AHA) Guidelines [15]. The secondary objective was to compare children’s to adults’ performances.

## Methods

### Study setting and participants

This quasi-experimental study took place in an elementary school located in Quebec City (Canada). All fifth and sixth graders (10 to 12 years old) were divided into four groups of 20 to 25 subjects and received CPR training at different times of the academic year. The groups and the training calendar were designed to fit the constraints of the school year as the CPR course was integrated into mandatory extra-curricular activities.

We recruited a comparison group of adult volunteers using convenience sampling. Volunteers were selected from two workplace CPR courses administered by the same instructors who taught the children group in our study. This sample was composed of truck drivers, administrative assistants, security agents, office workers, daycare educators, laboratory technicians, and gardeners.

### Training and assessment

Prior to any training, all participating schoolchildren were asked to answer an 11-item questionnaire about their personal characteristics and motivation to learn CPR. Thereafter, each group of children received a standardized 6-hour CPR course (three sessions of two hours each, over three weeks) based on the American Heart Association (AHA) 2005 Guidelines. Groups were trained between October 2006 and May 2007, using a similar training schedule. Each lesson was given on a Laerdal Little Ann manikin by two instructors. There were two students for one manikin and hands-on practice represented 60% of the course. Traditional CPR in-class training was chosen because it was by far the most frequently used teaching approach at the time, for both children and adults.

All participating children were asked to perform four cycles of CPR steps on a Skillreporter manikin (Laerdal) two or three days after the end of their own group training. Immediately before their performance, they were provided with standardized verbal instructions, following the assessment procedures developed by Brennan et al. 1996 [16].

Data on chest compressions (depth and rate) and volume of insufflations were retrieved from the Laerdal PC Skillreporting System. Video recordings were done for each performance and analyzed *a posteriori* by two independent assessors to determine to which extent the children were able to complete the right sequence of actions.

The adults were trained either in October 2007 or April 2008. They received a 6-hour CPR course similar to what the children had received. They were recorded on video at the end of their last CPR session and performed CPR on the same Skillreporter manikin. The same two assessors evaluated their performance independently.

### Outcomes

The primary outcome measure was the depth of chest compressions. Following the 2005 AHA Guidelines [15], adequate depth was defined as thoracic depression between 38 and 51 mm. Since our research question was to determine if elementary school students have the minimal required capacity to efficiently administer CPR to an adult, the capacity to reach the inferior threshold of 38 mm was the primary research focus.

The secondary outcome measures included compression rate, volume of insufflations, and CPR sequence of actions. As per the same AHA Guidelines, adequate rate was defined as a rhythm between 90 and 110 compressions per minute and adequate volumes were insufflations between 500 to 1000 ml. Again, the lower thresholds were the main targets of our analysis.

Finally, respect of the sequence of actions was measured using a previously validated instrument, including a 13-item checklist and a subjective overall performance rating scale (outstanding, very good, competent, questionably competent, not competent) [16]. The 1996 checklist was slightly modified to comply with the 2005 standards.

For each outcome measure, the schoolchildren’s performances were first compared to the AHA Guidelines standards and then to the adults’ performances.

### Independent assessors

The two independent assessors were two Emergency Medicine PGY-4 residents. For data analysis purpose, one was identified as the main evaluator and the other was used to calculate the inter-observer agreement to appraise the reliability of their evaluations. They were trained in the use of the standardized checklist and the subjective performance rating scale in August 2009. All videos and performances were then viewed and assessed from September to December 2009. The assessors were not involved in the design or data analysis for this study.

### Ethics

The study received ethics approval from the Université Laval Research Ethics Board (2006–206 A-1 R-1). Parental, student, and adult written consents were obtained for all subjects after providing precise written explanations about the study. All study forms and questionnaires were completed anonymously.

### Statistical analysis

The primary and secondary outcome measures are reported as continuous variables, except for the CPR sequence assessment that is reported as binomial categorical variables. We dichotomized the outcome measures provided by the Skillreporter manikin into adequate and inadequate categories according to the previously described AHA thresholds. For statistical comparisons between children and adults, we collapsed the five different overall performance-rating categories into two categories: competent (outstanding, very good, competent) and not competent (questionably competent and not competent).

The performance assessment required each participant to administer ten mouth-to-mouth insufflations. The Skillreporter manikin did not record insufflation attempts when there was no airflow through the lungs. This resulted in real, but unsuccessful attempts being made during the final evaluation. Therefore, we assigned a value of 0 ml to all missing breaths assuming that ten insufflations had been tried by each participant and we conducted a sensitivity analysis by calculating a worst-case scenario to determine the impact of this limitation of the manikin on the results.

We report means with 95% confidence intervals and used student’s t-tests (one-sided) with α = 0.05 to compare children to the AHA standards. We performed one-sided t-tests because we specifically wanted to know if children could exceed the minimum associated thresholds for each variable.

The adult sample size was small (n = 20) and not normally distributed for depth, volume and rate variables. As a result, we report medians, interquartile ranges (IQR) and Wilcoxon rank-sum tests to compare the outcome measures between children and adults. We used a two-group proportion Z-test to assess significant differences between adults and children for the subjective overall assessment and the sequence of actions. A p-value < 0.05 (two sided) was specified as the criterion of significance for all comparisons between children and adults. There was no *a priori* power calculation as both child and adult groups were samples of convenience. All analyses were performed using Stata Version 12.

## Results

Of the eligible 83 children in grades five and six, only one refused to participate in the study. Among the 82 students included in the study, two were absent when the evaluation was conducted on the manikin, for a total sample of 80 children included in the final analyses. Twenty adults were recruited for the comparison group on a voluntary basis.

Our initial cohort of 82 children was composed of 47 boys and 35 girls between the ages of 10 and 12 years, with a mean age of 10.6 (+/− 0.5) years. More than two-thirds were highly motivated to learn CPR and only a minority of these children had undergone some sort of CPR training prior to the beginning of this study (Table 1). The adults were aged between 18 and 60 years old and were taking a CPR course to become first aid officers within their workplaces.

**Table 1:** Children demographics

	
Gender – M(%)/F(%)	47(57.3)/35(42.7)
Age – mean (SD*)	10.6 (0.5)
Family members with heart diseases – n (%)	17 (21.3)
CPR training before the study – n (%)	6 (7.4)
Motivation to learn CPR	
High – n (%)	55 (67.1)
Moderate – n (%)	22 (26.8)
Low – n (%)	5 (6.1)

*Standard deviation.

**Figure 1:**
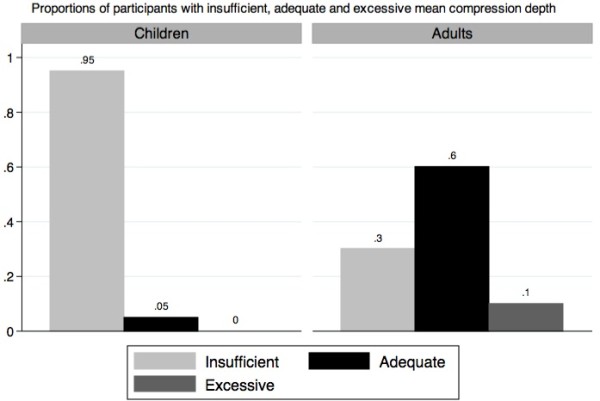
Compression depth in children and adults.

When performance was compared to the 2005 AHA Guidelines (Table 2), children did not achieve the minimum standards for compression depth (p = 1.00). Moreover, only 5.0% (0.1-9.9%) of them succeeded in performing CPR throughout the four cycles with a mean compression depth of at least 38 mm (Figure 1). Conversely, as a group, their mean compression rate reached the minimal recommended AHA standard (p < 0.001). Indeed, 92.5% (86.6-98.4%) of the children achieved a compression rate of more than 90/min during the evaluation.

**Table 2:** Performance of children compared to AHA guidelines

	**Minimum standard**	**Children: mean**	**95%CI**	p-value*
Depth (mm)	> 38	28.1	26.7-29.4	1.00
Rate (per min)	> 90	113.9	109.8-117.9	< 0.001
Volume (ml)	> 500	558.6	504.7-612.6	0.02
Volume (ml) Worst-case scenario	> 500	367.8	300.0-435.6	0.99

*One-sample *t*-test one sided significance level of 0.05.

**Figure 2:**
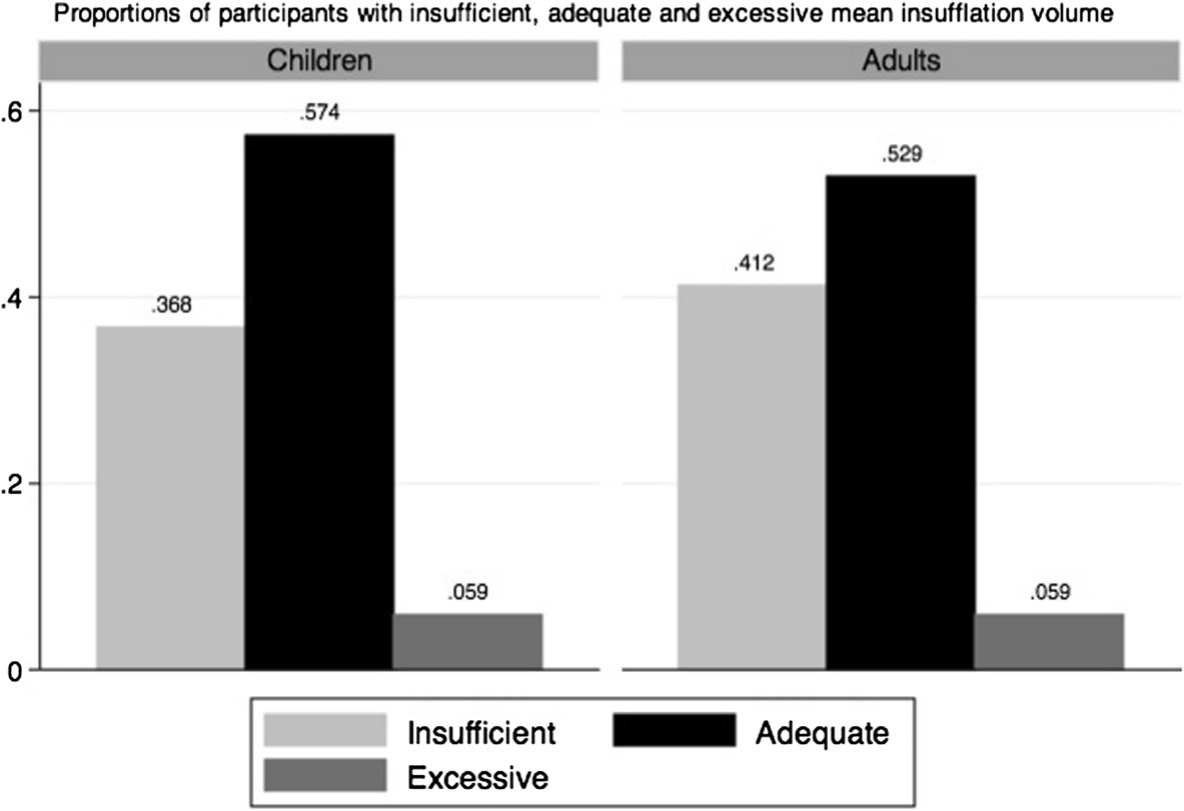
Insufflation volume in children and adults (actual data).

**Figure 3:**
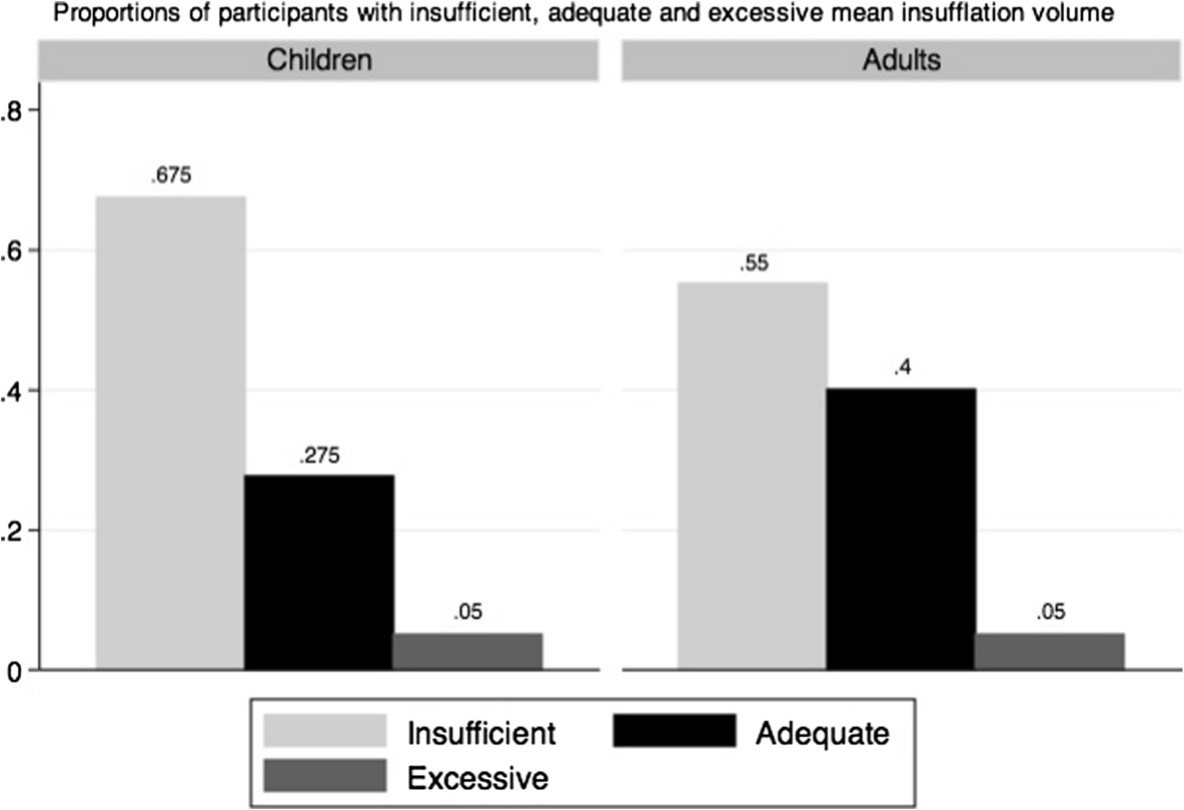
Worst-case scenario: insufflation volume in children and adults.

Twelve children and three adults did not have any ventilation recorded during their performance. Among the children who succeeded to administer at least one insufflation to the manikin, the mean ventilation volume was significantly greater than 500 ml (p = 0.02), while 63.2% of them (51.5-75.0%) provided rescue breathing over the minimal AHA threshold (Figure 2). However, the sensitivity analysis yielded very different results. When assigning 0 ml to all missing insufflations (worst-case scenario), the children’s mean insufflation volume did not reach the AHA guideline for rescue breathing (p = 0.99): only 32.5% (22.0-43.0%) of them provided a mean insufflation volume higher than 500 ml (Figure 3).

The median depth of thoracic compressions applied by the children was 28.0 mm (IQR: 7.5 mm), as compared to 43.5 mm (IQR: 11 mm) for the adults (Table 3), which represents a statistically significant difference (p < 0.001). The children performed CPR compressions at a median rate of 113.5 compressions/minute (IQR: 33 compressions/min) as compared to 109 compressions/minute (IQR: 30.5 compressions/min) for the adults (p = 0.47). The comparison of children to adults for the insufflation volume did not yield a significant difference, either with the actual data or the sensitivity analysis.

**Table 3:** Comparison of children to adults on depth and rate of compressions, and volume of insufflations

	**Children: median (IQR)**	**Adults: median (IQR)**	p-value*
Depth (mm)	28.0 (7.5)	43.5 (11.0)	< 0.001
Rate (per min)	113.5 (33.0)	109.0 (30.5)	0.47
Volume (ml)	548.5 (238.7)	520.0 (303.3)	0.83
Volume (ml) Worst-case scenario	348.0 (478.0)	457.5 (451.6)	0.48

*Wilcoxon Rank Sum Test and a two-sided significance level of 0.05.

**Figure 4:**
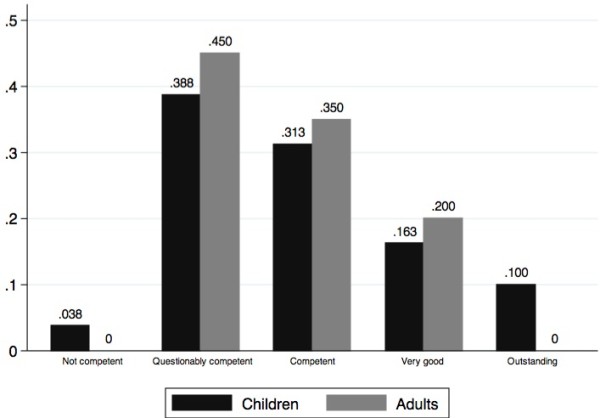
Fractional distribution of subjective overall performance in children and adults.

When collapsing the five categories of the overall performance assessment (Figure 4) into competent (outstanding, very good, competent) and not competent (questionably competent and not competent), 57.5% (45.9-68.5%) of children were assessed as competent, as compared to 55% (31.5-76.9%) of adults (p = 0.84). As for their sequential evaluation (Table 4), children were comparable to adults, except for step 1 (children better) and step 10 (adults better). Kappa statistic showed moderate to excellent inter-rater agreement on 11 of the 13 steps of the sequence. Assessment of Step 6 (Locates CPR hand position) and step 10 (Repeats 3 cycles of compressions) yielded a fair agreement between the two evaluators.

**Table 4:** Comparison of the proportions of correct completion of CPR steps among children and adults*

***Steps***	***Critical performance steps***	***Children- % (95%CI)***	***Adults - % (95%CI)***	***p-value***	***Kappa (95%CI)***
1	Checks for response	85.0 (77.0-93.3)	55.0 (31.1-78.9)	0.003	0.83 (0.65-1.00)
2	Activates EMS/ambulance	55.0 (43.9-66.1)	60.0 (36.5-83.5)	0.69	0.79 (0.67-0.91)
3	Opens airway	72.5 (62.5-82.5)	55.0 (31.1-78.9)	0.13	0.84 (0.70-0.94)
4	Checks breathing 3 seconds	62.5 (51.7-73.3)	50.0 (26.0-74.0)	0.31	0.72 (0.55-0.88)
5	Gives 2 breaths	66.3 (55.7-76.8)	55.0 (31.1-78.9)	0.35	0.83 (0.70-0.96)
6	Locates CPR hand position	96.3 (92.0-100.0)	95.0 (84.5-100.0)	0.80	0.31 (−0.19-0.81)
7	Delivers 25–35 compressions	96.3 (92.0-100.0)	100.0	0.38	0.52 (0.16-0.88)
8	Opens airway	76.3 (66.7-85.8)	85.0 (67.9-100.0)	0.40	0.68 (0.48-0.88)
9	Gives 2 breaths	75.0 (65.3-84.7)	75.0 (54.2-95.8)	1.0	0.82 (0.68-0.97)
10	Repeats 3 cycles of compressions	80.0 (71.0-89.0)	100.0	0.03	0.33 (0.12-0.54)
11	Opens airway	66.3 (55.7-76.8)	80.0 (60.8-99.2)	0.23	0.53 (0.34-0.73)
12	Gives 2 breaths between the compressions cycles	61.3 (50.3-72.2)	65.0 (42.1-87.9)	0.78	0.81 (0.67-0.94)
13	Locates CPR hand position for each cycle	91.3 (84.9-97.6)	100.0	0.17	0.64 (0.32-0.97)

* Two-group proportion Z-test at a two-sided significance level of 0.05.

## Discussion

In this quasi-experimental study, we assessed if children 10 to 12 years of age were able to achieve the minimal 2005 AHA recommended standards for compression depth and rate, and volume of insufflation. Only 5.0% of the students reached a mean compression depth of 38 mm. The mean compression depth for all children was 28.1 mm (26.7-29.4). Conversely, when insufflation attempts were captured by the manikin, the children’s mean rescue-breathing volume did attain the recommended lower limit for volume of insufflations. Similarly, 92.5% of schoolchildren achieved a mean compression rate equal to or greater than 90/minute and complied with all sequence steps 60% of the time, except for calling for help.

To our knowledge, Jones et al. 2007 is the only other article reporting on measures of compression depth and rate for elementary school students [11]. In their study, no children aged 9–10 years old were able to perform CPR with adequate chest compression depth between 38 and 51 mm, while 19% of pupils 11–12 years of age and 45% of those aged 13–14 reached an adequate mean compression depth. For the same three age groups, the mean compression rates were respectively 108 (100–116), 109 (102–116) and 116 (109–123) compressions per minute. They subsequently stated that their pupils aged 13–14 years achieved CPR performances comparable to adults in other studies. In our own study, 5% (0.1-9.9%) of the students aged 10–12 years and 60% (35.5-83.5%) of the adults in the convenience sample provided chest compressions in the adequate depth range (38–51 mm). The children’s mean compression rate was 113.9 per minute (109.8-117.9). Our findings support their results.

CPR training during our project followed the adult 2005 AHA Guidelines for compression depth, which defined adequate depth as thoracic depression between 38 and 51 mm [15]. Those standards have changed since the completion of our study and the 2010 AHA Guidelines on CPR now state that compression depth should reach at least 50 mm [17]. However, this new standard does not change our results since no children had a mean compression depth over 50 mm.

In the same 2010 AHA Guidelines, rescue breathing has been significantly demoted, but is still recommended for trained rescuers [17]. No other studies have previously published volume measures for elementary schoolchildren. In this study, the sensitivity analysis (including the presumed 0 ml insufflations) may better represent the true distribution of rescue-breathing volume, since most participants tried to administer the required 10 insufflations. In this scenario, only 32.5% (22.0-43.0%) of the children provided a mean volume of ventilation higher than 500 ml. However, we did not find a significant difference between children’s and adults’ performances. Moreover, our adult group achieved rescue-breathing volume similar to adults from other studies [18,19].

Previous studies have focused on assessing CPR sequence knowledge acquisition in elementary schoolchildren at diverse ages. Bollig et al. 2009 and Uray et al. 2004 have shown that pupils as young as 6–7 years old are capable of learning basic life support interventions after school-based courses [20,21]. Lubrano et al. 2005 and Connolly et al. 2007 arrived at similar conclusions with children aged 8–11 and 10–12 years [13,22]. However, in studies including elementary school students, only Lester et al. 1996 has reported a practical assessment of each CPR step performed by children 11–12 years of age after three 1-hour sessions [14]. Their cohort of 31 students clearly underperformed for certain CPR actions as compared to the pupils in our study. More specifically, call for help or ambulance (12.9%, 95%CI 3.6-29.8% performed) and airway opening (22.6% 95%CI 9.6-41.1% performed) were particularly divergent from our results. In our study, schoolchildren adequately called for help or an ambulance 55.0% of the time (43.9-66.1%). By adding the participants who did call the ambulance at the wrong point in the recommended sequence, the proportion of those who called for help or ambulance rises to 76.3% (66.7-85.8%). Shorter length of training, longer delay between last training session and practical assessment (9 days), and a different rating scale are the most obvious reasons that could explain such heterogeneity in results.

In regards to the sequence of CPR and subjective overall performance, we did not identify differences between our student and adult groups. Moreover, our students’ performance for each CPR step and overall assessment scale is comparable to adults’ performance in previous CPR studies [18,19,23].

There are a number of potential limitations to our study. Sample size and lack of power calculation limit the generalizability of our conclusion that there is no difference between children and adults for all non-significant comparisons. Also, we did not measure weight and height as potential factors influencing children’s performance. In Canada, median weights for girls and boys 9 to 13 years of age are 42.5 and 43.0 kg, respectively [24]. That being said, our study was meant to contribute to the debate on the optimal age for introducing CPR into school curriculum. From a school perspective, implementation of a CPR training program would most likely be determined by age groups and grades, not by height and weight of each individual.

**Figure 5:**
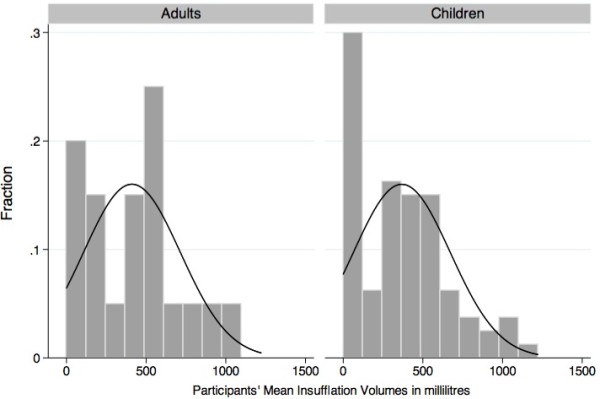
Worst-case scenario: distribution of mean insufflation volumes in adults and children.

Another important limitation of our analyses is related to the insufflations not recorded by the manikin, as previously discussed. It is impossible to state if the missing insufflations were the consequence of either a poor airway opening, a true insufficient strength to insufflate, an omission or a technical issue related to the manikin manipulation. However, when looking at the distribution of the participants’ mean volumes of insufflations from the sensitivity analysis (Figure 5), we notice a bimodal distribution with 0 ml as one of the modes. This fact leads us to think two different populations are captured in this distribution and insufficient strength may not be the only explanation for low volume. We hypothesize an inefficient or partial airway opening explains a large number of 0 ml insufflations. However, the most important outcome remains the administration of inefficient insufflations and we believe our conclusions must take that into account.

## Conclusions

In this study, schoolchildren of 10–12 years old did not achieve chest compressions that reached the AHA lower threshold. When children succeeded in providing insufflations to the manikin, their mean volume of insufflations was sufficient, but the large number of unsuccessful rescue-breathing attempts may have led to overall inefficient breathing support. Conversely, they achieved an acceptable compression rate and a significant proportion of them were able to learn and competently perform CPR steps and sequence. Systematically training this age group would more likely lead to knowledge acquisition than to efficient CPR administration according to the AHA standards.

## Abbreviations

AHA: American heart association; CPR: Cardiopulmonary resuscitation; EMS: Emergency medical services; PGY: Post-graduate year; Kg: Kilograms.

## Competing interests

The authors have no conflicts of interest and no financial relationships to disclose.

## Authors’ contributions

SB, MP, IB, AB, MMC, RL and NL conceptualized and designed the study. SB, MP, IB, AB and MMC designed the data collection instruments and supervised data collection. SB and MP trained the CPR performance assessors. EBP and MSO assessed participants’ CPR performance. SB and SC carried out the statistical analyses. SB, MP and IB drafted and revised the initial manuscript. All authors read and approved the final manuscript.
